# Infestation by the Piercing–Sucking Herbivore *Nilaparvata lugens* Systemically Triggers JA- and SA-Dependent Defense Responses in Rice

**DOI:** 10.3390/biology12060820

**Published:** 2023-06-05

**Authors:** Heng Li, Liping Xu, Weiping Wu, Weizheng Peng, Yonggen Lou, Jing Lu

**Affiliations:** State Key Laboratory of Rice Biology & Ministry of Agriculture Key Lab of Agricultural Entomology, Institute of Insect Sciences, Zhejiang University, Hangzhou 310058, China; sdauliheng@163.com (H.L.); 21616161@zju.edu.cn (L.X.); 3170100432@zju.edu.cn (W.W.); 3170100205@zju.edu.cn (W.P.)

**Keywords:** herbivore-induced plant defense, local defense, systemic defense, marker genes, defense-related signaling pathway

## Abstract

**Simple Summary:**

Previous studies have proved that brown planthopper (BPH), *Nilaparvata lugens*, a piercing–sucking herbivore, activates strong local defenses in rice. However, whether a BPH infestation elicits systemic responses in rice remains largely unknown. By detecting the change in transcript levels of 12 jasmonic acid (JA) and/or salicylic acid (SA) signaling-responsive marker genes in different tissues of rice plants upon a BPH attack, we demonstrate that an infestation of gravid BPH females systemically activates JA- and SA-dependent defenses in rice, which may in turn influence the composition and structure of the community in the rice ecosystem.

**Abstract:**

It has been well documented that an infestation of the piercing–sucking herbivore, brown planthopper (BPH), *Nilaparvata lugens*, activates strong local defenses in rice. However, whether a BPH infestation elicits systemic responses in rice remains largely unknown. In this study, we investigated BPH-induced systemic defenses by detecting the change in expression levels of 12 JA- and/or SA-signaling-responsive marker genes in different rice tissues upon a BPH attack. We found that an infestation of gravid BPH females on rice leaf sheaths significantly increased the local transcript level of all 12 marker genes tested except *OsVSP*, whose expression was induced only weakly at a later stage of the BPH infestation. Moreover, an infestation of gravid BPH females also systemically up-regulated the transcription levels of three JA-signaling-responsive genes (*OsJAZ8*, *OsJAMyb*, and *OsPR3*), one SA-signaling-responsive gene (*OsWRKY62*), and two JA- and SA- signaling-responsive genes (*OsPR1a* and *OsPR10a*). Our results demonstrate that an infestation of gravid BPH females systemically activates JA- and SA-dependent defenses in rice, which may in turn influence the composition and structure of the community in the rice ecosystem.

## 1. Introduction

Plants and herbivorous insects on earth have coexisted for at least 350 million years. To protect themselves from herbivory, plants have evolved sophisticated mechanisms, including constitutive defenses and herbivore-induced plant defenses [1,2,3]. Herbivore-induced plant defenses occur when plants specifically recognize molecular patterns associated with herbivore damage and, in response, initiate early signaling events. These early signaling events include the depolarization of the membrane potential, the increase in the level of cytosolic calcium ions, the burst of reactive oxygen species, and the activation of mitogen-activated protein kinase (MPK) cascades [4,5,6]. The activation of early signaling events triggers the pathways mediated by defensive phytohormones, mainly jasmonic acid (JA), salicylic acid (SA), and ethylene. The activated signaling pathways cause changes in the transcriptome and metabolome of plants that enhance their resistance to herbivores [2,6,7].

From the perspective of space, herbivore-induced plant defenses not only occur at the attacked sites (locally, referred to as local defenses) but also extend to non-attacked distal tissues (systemically, referred to as systemic defenses) [8,9,10]. Therefore, herbivore infestation could systemically enhance the resistance of plants to herbivores sharing the same plants, including conspecifics or heterospecifics. For example, foliar feeding by the generalist *Spodoptera littoralis* reduces the colonization and growth of larvae of the root feeder western corn rootworm (WCR, *Diabrotica virgifera virgifera*) by altering direct and indirect defensive compounds in maize roots, especially when the foliar herbivory is prior to the root feeding [11]. Root herbivory by *D. v. virgifera*, in turn, enhances levels of 2,4-dihydroxy-7-methoxy-1,4-benzoxazin-3-one (DIMBOA) and primes the production of chlorogenic acid (CGA) in maize shoots, which increases the resistance of maize shoots to *S. littoralis* [12]. The infestation of *Helicoverpa armigera* larvae results in a significant inhibition and repellent effect of cotton plants on the populations of *Aphis gossypii* by increasing the accumulation of JA and gossypol and tannins [13]. Although herbivore-induced systemic defenses have been reported in many plant species, the activated systemic responses in plants not only change with species and density of herbivores [14], but also with the development stage and tissues/organs of plants [15,16]. In *Nicotiana attenuata*, for example, the defoliator (*Manduca sexta*) systemically elicits the accumulation of JA in both attacked leaves and not-attacked stems, whereas the stem borer (*Trichobaris mucorea*) only activates JA signaling in local stems but not in systemic leaves [17]. In addition, the diurnal rhythms of generalized and specialized metabolisms are quite different in attacked local leaves and not-attacked systemic leaves and roots of tobacco plants infested by *M. sexta*, indicating that *M. sexta*-induced systemic defense responses in tobacco are highly tissue-specific [18]. Similarly, mimicking shoot herbivory with exogenous treatments with JA elicits systemic molecular and chemical responses in the roots and shoots of *Brassica oleracea* are different from those induced by treating roots with JA (mimicking root herbivory) [16].

Investigating the change in transcript levels of marker genes related to plant defenses in different tissues of plants can clarify whether plant defenses, and which defensive pathways, are locally and systemically activated by herbivore infestations. Moreover, these changes may also explain some biological phenomena. In *Arabidopsis thaliana*, for example, *VSP2* (*VEGETATIVE STORAGE PROTEIN 2*) and *PDF1.2* (*PLANT DEFENSIN 1.2*) are the marker genes related to the activation of the JA-MYC branch and JA-ERF branch, respectively. As *Pieris rapae* larvae prefer to feed on JA-ERF branch-activated plants and an infestation of *P. rapae* locally induces the expression of *VSP2* but suppresses *PDF1.2*, plants can activate the JA-MYC branch to prevent the activation of the JA-ERF branch, thereby reducing the attractiveness of the plant to *P. rapae* [19]. In *Arabidopsis* roots, the transcript level of the JA- and ethylene-signaling marker gene *PR4* is systemically up-regulated upon infestation by the thrip (*Frankiniella occidentalis*) but not by the spider mite (*Tetranychus urticae*), suggesting that the thrip-induced plant susceptibility to the root cyst nematode *Heterodera schachtii* is due to the activation of the JA and ethylene pathway in roots [20]. The transcript level of two SA pathway-associated marker genes, *PHNYLALANINE AMMONIA LYASE* (*PAL*) and *PATHOGENESIS-RELATED1* (*PR1*), is up-regulated in the roots of *B. oleracea* from an infestation of one of two above-ground herbivores, a leaf-chewing diamondback moth caterpillar (*Plutella xylostella*) and a phloem-feeding cabbage aphid (*Brevicoryne brassicae*). The results indicate that both herbivores systemically activate the SA signaling pathway in *B. oleracea* [21]. Recently, Liu et al. (2022) have demonstrated that when the defoliator *Plagiodera versicolora* feeds on poplar, a systemic defense is activated: transcript levels of the two JA pathway-marker genes, *JA ZIM-domain* (*JAZ*) and *CORONATINE INSENSITIVE 1* (*COI1*), are up-regulated in adjacent undamaged leaves of poplar [22]. These results suggest that marker genes are potent molecular tools for elucidating the mechanisms underlying herbivore-induced plant defenses.

Rice (*Oryza sativa*), one of the most important food crops in the world, suffers from many insect pests and pathogens, including the brown planthopper (BPH), *Nilaparvata lugens* [23]; white-backed planthopper (WBPH, *Sogatella furcifera*) [24]; striped stem borer (SSB, *Chilo suppressalis*) [25]; and leaf folder (LF, *Cnaphalocrocis medinalis*) [26]. The BPH damages rice plants mainly by sucking sap of the phloem, laying eggs in leaf sheaths, and transmitting viruses, which causes a decrease in nitrogen concentrations, chlorophyll contents and photosynthetic rate, and disruption of vascular bundles, resulting in a yield loss and low quality of rice [27]. In China, the yield loss of rice caused by the BPH is more than 2 million tons per year [28]. To date, some common marker genes related to rice defenses against pathogens have been reported. Genes—for instance, *OsJAZ8* [29], *OsJAMyb* [30], *OsPR3* [31], *OsPR4* [32], and *OsVSP* [33]—are regarded as JA-pathway-responsive genes; *OsPAL* [34] and two WRKY genes, *OsWRKY45* and *OsWRKY62* [35,36], are reported to be marker genes that activate the SA pathway. Moreover, four PR genes, including *OsPR1a* [37], *OsPR1b* [38], *OsPR10a* [39], and *OsPR10c* [40], are responsive to both JA and SA pathways. A BPH infestation regulates signaling pathways mediated by JA, SA, ethylene, abscisic acid (ABA), and hydrogen peroxide in attacked leaf sheaths of rice plants, and these signaling pathways in turn change the transcriptome and metabolome of plants and regulate the direct and indirect resistance of rice to BPHs [41,42,43,44]. Moreover, some genes that are involved in rice defenses, such as *OsHI-LOX* (*13-LIPOXYGENASE*) [45] and *OsWRKY45* [46], have been reported to be induced by a BPH infestation. However, the genes that could be used as marker genes related to BPH-induced rice defenses remain largely unknown. In addition, it remains unclear whether rice defenses are systemically induced by a BPH infestation, and if so, which pathways are involved.

To address the above issues, in this study, first, we decided which genes could be used as marker genes for the JA- and SA-pathways activated by a BPH infestation in rice, and then, we assessed whether the BPH infestation could systemically induce defense responses mediated by these pathways. We demonstrate that an infestation of gravid BPH females systemically activates defense responses mediated by JA- and SA-pathways in rice.

## 2. Materials and Methods

### 2.1. Plants

The rice variety used in this paper was Xiushui 110 (Japonica). Rice seeds were soaked in water for 24 h, and then, they were transferred into a petri dish containing moist filter paper. The seeds were germinated in an incubator (28 ± 2 °C temperature, with a photoperiod of 14 h light: 10 h dark). Ten-day-old seedlings were transferred to rectangular hydroponic boxes (length 45 cm, width 35 cm, height 17 cm) with nutrient solution [44] in phytotron (28 ± 2 °C temperature, with a photoperiod of 14 h light: 10 h dark, 50–65% relative humidity). Individual four-week-old (five-leaf stage) seedlings were transplanted to 350-mL plastic pots, and 4 days later, plants were used for experiments.

### 2.2. Insects

BPH colonies were collected from paddy fields in Hangzhou, Zhejiang province, China, and raised on Taichung native (TN1, a rice variety susceptible to BPH) seedlings in phytotron (27 ± 2 °C temperature, with a photoperiod of 14 h light: 10 h dark, 60–65% relative humidity). Newly emerged adults (the ratio of females to males was 2:1) were transferred to fresh TN1 seedlings for feeding, mating, and oviposition; 4 days later, gravid BPH females were collected for experiments.

### 2.3. Plant Treatments and Sample Harvesting

**Figure 1:**
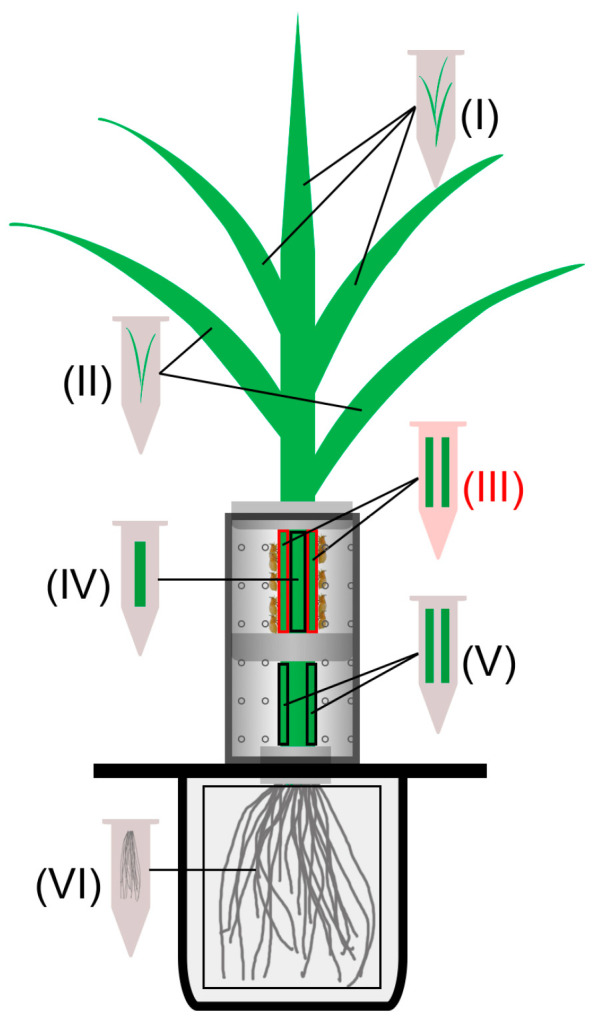
Schematic diagram of tissues harvested from BPH-infested rice; 10 gravid BPH females were introduced into the upper part of the cylinder and were allowed to feed and oviposit on rice. For BPH-infested plants, inner leaf blades linked to uninfested leaf sheaths (LB-ULS, part I), leaf blades linked to infested leaf sheaths (LB-ILS, part II), damaged parts of infested leaf sheaths (DP-ILS, part III; marked in red), inner uninfested leaf sheaths (ULS, part IV), undamaged parts of infested leaf sheaths (UP-ILS, part V), and roots (part VI) were separately harvested after BPH inoculation. The same parts of rice plants with empty cylinders were harvested as controls.

XS110 plants were randomly divided into BPH-infested and uninfested (control) groups. For BPH infestation, rice plants were individually confined in glass cylinders (diameter 4 cm, height 9 cm, with 48 air holes) (the top and middle of the cylinder were separately sealed with a round sponge), into which 10 gravid BPH females were introduced into the upper part of the cylinder (shown in Figure 1). Plants with the same empty cylinders and sponge were used as controls.

Given that BPH females mainly feed and lay eggs on the leaf sheaths of the outermost two leaves, six parts of infested plants were separately harvested at 3, 12, 24, 48, and 72 h after BPH infestation (Figure 1). These six parts included inner leaf blades linked to uninfested leaf sheaths (LB-ULS, part I), leaf blades linked to infested leaf sheaths (LB-ILS, part II), damaged position of infested leaf sheaths (DP-ILS, part III), uninfested leaf sheaths (ULS, part IV), undamaged position of infested leaf sheaths (UP-ILS, part V), and roots (part VI). The same parts of uninfested (control) plants were also harvested. Each treatment at each time point was replicated six times.

### 2.4. Quantitive Real-Time PCR (qRT-PCR)

For qRT-PCR analysis, freshly harvested rice tissues were ground into fine powder in liquid nitrogen, and 100 mg samples were used for total RNA isolation using the SV Total RNA Isolation System (Promega, Madison, WI, USA) following the modified manufacturer’s instructions. The concentration and purity of RNA were detected using the BioDrop Spectrophotometer (Biochrom, WC, UK).

RNA (500 ng) was reverse-transcribed using PrimeScript^TM^ RT PCR Kit (TaKaRa, Dalian, China). An amount of 1 μL of cDNA was used for qRT-PCR assay using the SosoFast^TM^ Probes Supermix (Bio-Rad, Hercules, CA, USA), and the reaction was carried out on CFX96^TM^ Real-Time System (Bio-Rad). All procedures were performed according to the manufacturer’s instructions. The expression profiles of twelve candidate genes that related to JA- or/and SA-signaling pathways, including *OsHI-LOX* (Os08g39840), *OsPAL* (Os02g41680), *OsJAZ8* (Os09g26780), *OsWRKY62* (Os01g51690), *OsVSP* (Os01g09540), *OsJAMyb* (Os11g45740), *OsPR3* (Os05g33130), *OsPR4* (Os11g37970), *OsPR1a* (Os07g03710), *OsPR1b* (Os01g28450), *OsPR10a* (Os12g36880), and *OsPR10c* (Os03g18850) were investigated. The rice actin gene *OsACT* (Os03g50885) was used as an internal standard to normalize cDNA concentrations in tested genes. The transcript level of each gene was determined from threshold cycle (CT) values according to the standard curve, and the relative expression level of target genes was calculated by dividing the transcript level of the target gene by the transcript level of *OsACT* from the same sample. Primers and probes used for qRT-PCR assay are shown in Table S1. Each treatment at each time point was replicated six times.

### 2.5. Data Analysis

Data analysis was carried out with PASW Statistics 18.0 software (SAS Institute, Inc., Cary, NC, USA, http://www.sas.com/ (accessed on 4 October 2020)). Differences in transcription levels between two groups were analyzed with two-tailed independent sample Student’s *t*-test. The normality of data was tested using the Kolmogorov–Smirnov test (*p* < 0.05), and the equality of variances was tested by using Levene’s test (*p* < 0.05). In the case of unequal variances, the corrected *t*-value and corresponding *p*-value were used.

## 3. Results

### 3.1. BPH Infestation Locally Activates JA- and SA-Dependent Responses

**Figure 2:**
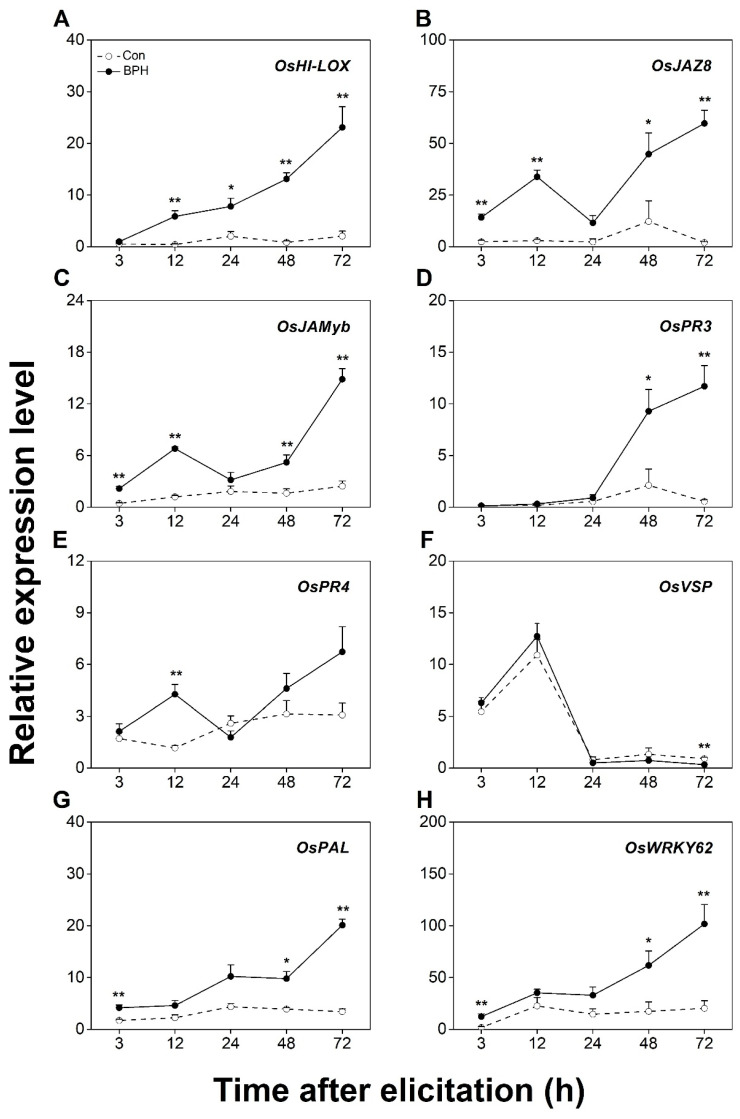
BPH infestation locally induces the expression of JA- or SA-signaling-related genes. Relative transcription levels (mean + SE, n = 6) of *OsHI-LOX* (**A**), *OsJAZ8* (**B**), *OsJAMyb* (**C**), *OsPR3* (**D**), *OsPR4* (**E**), *OsVSP* (**F**), *OsPAL* (**G**), and *OsWRKY62* (**H**) in leaf sheaths (part III in Figure 1) of rice plants with BPH infestation (BPH) or without (Con). Asterisks indicate significant differences between treatments (*, *p* < 0.05; **, *p* < 0.01; Student’s *t*-test).

A BPH infestation activates JA- and SA-mediated signaling pathways, both of which contribute to the subsequent resistance of rice to BPHs [42,47,48,49]. Therefore, to determine which marker genes are related to defenses induced by a BPH infestation, we first investigated changes in the transcript level of twelve candidate genes related to both signaling pathways at local BPH infestation sites (damaged position of infested leaf sheaths, DP-ILS, part III in Figure 1). The transcript level of the JA biosynthesis-related gene *OsHI-LOX* was continually increased in BPH-infested leaf sheaths during 72 h of a BPH infestation (Figure 2A). Moreover, among the transcript levels of five JA-responsive genes—*OsJAZ8*, *OsJAMyb*, *OsPR3*, *OsPR4*, and *OsVSP* [29,30,31,32,33]—those of *OsJAZ8* were obviously induced by the BPH infestation at the damaged leaf sheaths (Figure 2B). The transcript level of *OsJAMyb* exhibited significant elevation at 3, 12, 48, and 72 h post-BPH infestation, whereas the expression levels of *OsPR3* and *OsPR4* were notably induced at late and early stages of the BPH infestation, respectively (Figure 2C–E). However, almost no change in *OsVSP* transcripts was observed in response to the BPH attack (Figure 2F). The BPH infestation also up-regulated the transcript level of *OsPAL* (Figure 2G), a SA biosynthesis-related gene [34], and *OsWRKY62* (Figure 2H), which encodes an SA-responsive core transcription factor at the infested sites [35,36].

**Figure 3:**
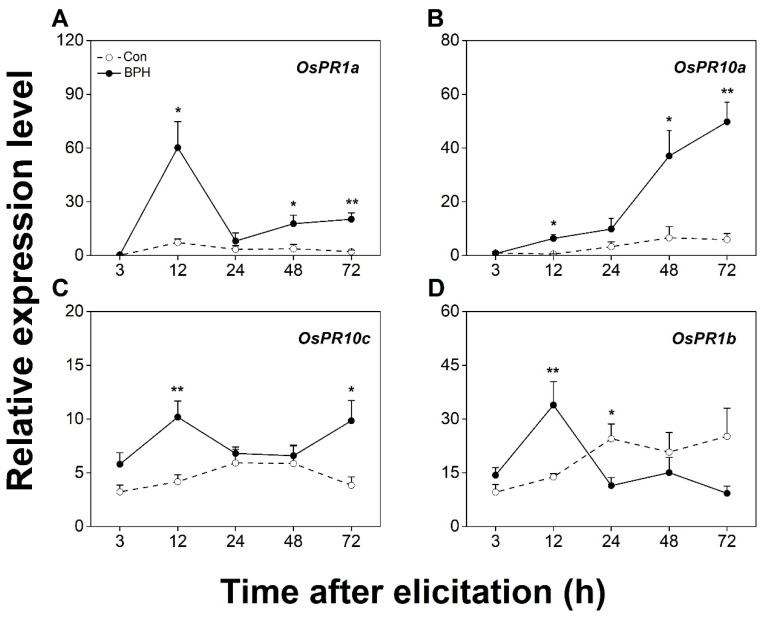
BPH infestation locally induces the expression of both JA- and SA-signaling-responsive genes. Relative transcription levels (mean + SE, n = 6) of *OsPR1a* (**A**), *OsPR1b* (**B**), *OsPR10a* (**C**), and *OsPR10c* (**D**) in leaf sheaths (part III in Figure 1) of rice plants with BPH infestation (BPH) or without (Con). Asterisks indicate significant differences between treatments (*, *p* < 0.05; **, *p* < 0.01; Student’s *t*-test).

The BPH infestation strongly elevated the transcript levels of JA/SA-responsive *OsPR1a*, *OsPR10a*, and *OsPR10c* in the infested tissues (Figure 3A–C). Interestingly, the expression of *OsPR1b* was up-regulated 12 h after the BPH attack, yet it was down-regulated 24 h later (Figure 3D). Based on these results, we selected JA-responsive *OsJAZ8*, *OsJAMyb*, and *OsPR3*; SA-responsive *OsWRKY62*; and JA/SA-responsive *OsPR1a* and *OsPR10a* as BPH-responsive marker genes for the following experiments.

### 3.2. BPH Infestation Systematically Activates JA- and/or SA-Dependent Responses in Rice

**Figure 4:**
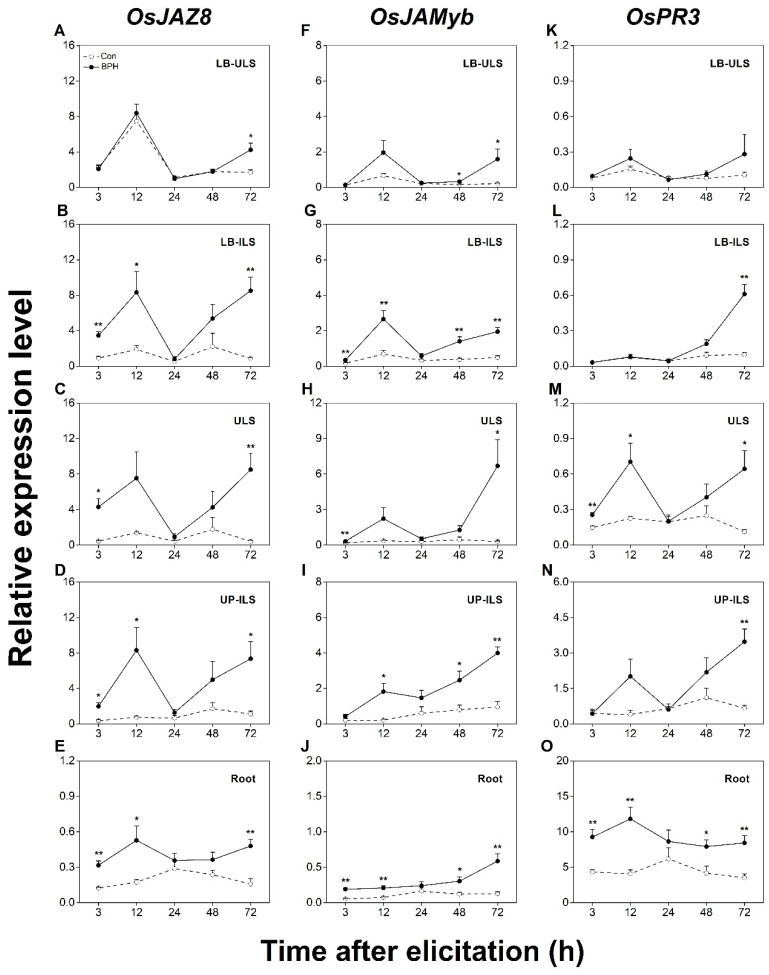
BPH infestation systemically induces the expression of JA- signaling-responsive genes. Relative transcription levels (mean + SE, n = 6) of JA-responsive genes *OsJAZ8* (**A**–**E**), *OsJAMyb* (**F**–**J**), and *OsPR3* (**K**–**O**) in indicated tissues of rice plants with BPH infestation (BPH) or without (Con). LB-ULS: inner leaf blades linked to uninfested leaf sheaths (part I in Figure 1); LB-ILS: leaf blades linked to infested leaf sheaths (part II in Figure 1); ULS: inner uninfested leaf sheaths (part IV in Figure 1); UP-ILS: undamaged parts of infested leaf sheaths (part V in Figure 1); root: part VI in Figure 1. Asterisks indicate significant differences between treatments (*, *p* < 0.05; **, *p* < 0.01; Student’s *t*-test).

To evaluate whether BPH induced systemic responses in rice, we divided BPH-infested and control plants into six different parts (Figure 1), which included leaf blades linked to infested leaf sheaths (LB-ILS), inner uninfested leaf sheaths (ULS), infested leaf sheaths (ILS), undamaged position of infested leaf sheaths (UP-ILS), leaf blades linked to uninfested leaf sheaths (LB-ULS), and roots (part VI). We first measured the change in transcript levels of the above JA-responsive marker genes in all systemic tissues. As shown in Figure 4, the constitutive transcript levels of *OsJAZ8* and *OsJAMyb* in rice roots were lower than those in leaf blades and leaf sheaths (Figure 4A–J). Just as the local induction patterns changed in response to the BPH infestation, the transcript levels of *OsJAZ8* and *OsJAMyb* were also significantly up-regulated in all tested systemic tissues, especially in the LB-ILS, ULS, and UP-ILS of infested plants (Figure 4B–D,G–I). In contrast, the transcript levels of *OsJAZ8* and *OsJAMyb* in the LB-ULS of infested plants increased only at later stages (Figure 4A,F), and the transcript levels of the two genes in roots were weakly induced (Figure 4E,J). Interestingly, the constitutive transcript levels of *OsPR3* in rice roots were highest in all measured tissues (Figure 4K–O). Although *OsPR3* was locally induced at a later stage of the BPH infestation (Figure 2D), its transcripts accumulated most rapidly in the ULS and roots of BPH-infested plants (Figure 4M,O). Moreover, the induced transcript level of *OsPR3* in DP-ILS was comparable to that in the roots (Figure 2D). The BPH infestation also resulted in a significant up-regulation of *OsPR3* transcripts in LB-ILS and UP-ILS at 72 h post-infestation (Figure 4L,N); however, it did not change the transcript level of *OsPR3* in LB-ULS at all tested time points (Figure 4K). These results clearly suggest that a BPH infestation systemically induced JA-dependent responses in rice.

**Figure 5:**
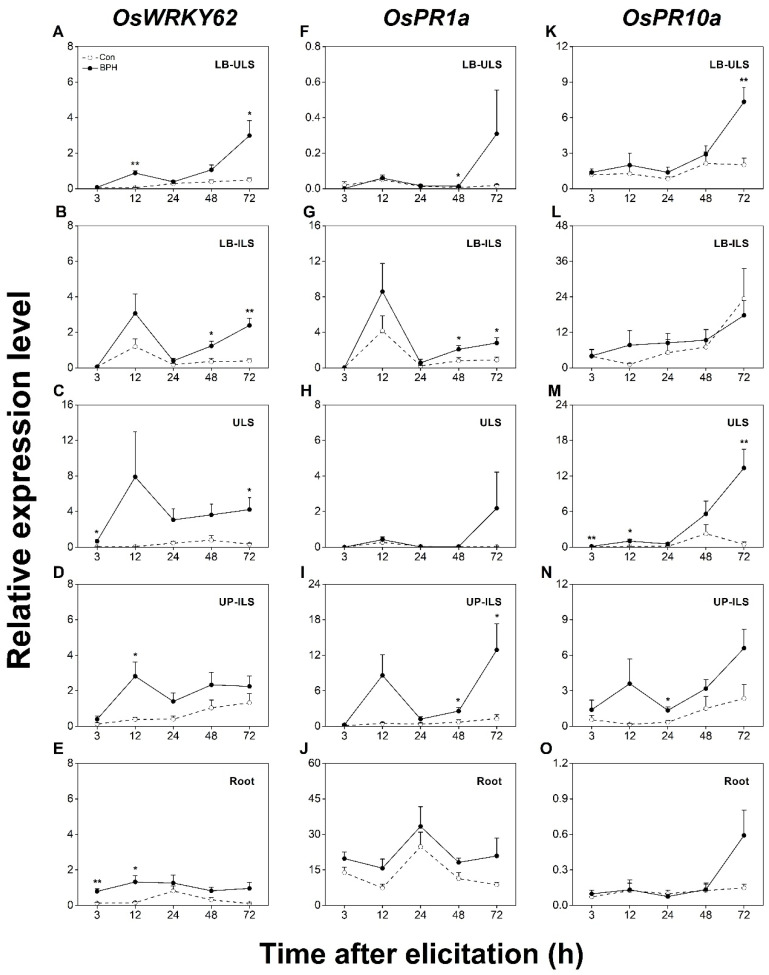
BPH infestation systemically induces the expression of SA signaling or both JA- and SA-signaling-responsive genes. Relative transcription levels (mean + SE, n = 6) of JA-dependent marker genes *OsWRKY62* (**A**–**E**), *OsPR1a* (**F**–**J**), and *OsPR10a* (**K**–**O**) in indicated tissues of rice plants with BPH infestation (BPH) or without (Con). LB-ULS: inner leaf blades linked to uninfested leaf sheaths (part I in Figure 1); LB-ILS: leaf blades linked to infested leaf sheaths (part II in Figure 1); ULS: inner uninfested leaf sheaths (part IV in Figure 1); UP-ILS: undamaged parts of infested leaf sheaths (part V in Figure 1); root: part VI in Figure 1. Asterisks indicate significant differences between treatments (*, *p* < 0.05; **, *p* < 0.01; Student’s *t*-test).

We next evaluated the systematic induction of SA- and JA/SA-dependent responses in rice by detecting changes in the transcript levels of *OsWRKY62*, *OsPR1a*, and *OsPR10a*. As shown in Figure 5, the constitutive transcript level of *OsWRKY62* was low; however, when plants were infested by BPH, the transcript level of *OsWRKY62* increased in all systemic tissues (Figure 5A–E). Like *OsPR3*, *OsPR1a* exhibited the highest constitutive transcript levels in rice roots in all tested tissues. Generally, *OsPR1a* was also systemically induced by BPH infestation, with the highest induction occurring in UP-ILS (Figure 5F–J). The constitutive transcript level of *OsPR10a* was similar in all tested tissues. When plants were attacked by BPHs, the transcript level of *OsPR10a* increased mainly in ULS (Figure 5M). Taken together, these data indicate a BPH infestation can also systematically activate SA- and JA/SA-dependent responses in rice.

## 4. Discussion

When infested by herbivorous insects, plants can protect themselves from invaders by activating a range of local and systemic defenses. These defenses may influence the performance of all organisms on a plant either simultaneously or in succession, thereby influencing the composition and structure of the community in the plant ecosystem. Therefore, it is important to elucidate which defenses can be systemically activated and which defenses can only be locally activated in a specific plant–herbivore system. In this study, by detecting the change in transcript levels of marker genes related to rice defenses in different tissues of rice plants, we demonstrate that an infestation of gravid BPH females systemically activates defenses mediated by JA- and SA-signaling pathways, and the systemic responses in rice induced by BPHs are tissue-specific.

Previous work has revealed that a BPH infestation activates JA- and SA-signaling pathways and their downstream defense responses and demonstrated that both pathways play crucial roles in regulating the resistance of rice to BPHs [41,42,43,44]. In line with these findings, we found in this study that a BPH infestation up-regulates the transcript level of JA and SA biosynthetic and responsive genes not only in BPH-infested leaf sheaths, but also in the undamaged tissues of BPH-infested plants, such as leaf blades and roots (Figure 2, Figure 3, Figure 4 and Figure 5). These data suggest that a BPH infestation systemically activates the JA- and SA-mediated signaling pathways in rice. It has been reported that a BPH infestation also activates ABA- and gibberellin-mediated signaling pathways [43,48,49]. Moreover, ABA and gibberellin pathways play an important role in regulating the resistance of rice to BPHs and other insect pests, such as WBPHs [47] and SSBs [50]. Therefore, further research should elucidate whether other BPH-elicited signaling-pathway-dependent defenses are also systemic and how these signaling pathways shape the systemic responses in rice induced by BPHs.

Herbivore infestations could influence the performance of all organisms on a plant, either simultaneously or in succession, by altering a plant’s physiological and biochemical status [3,10,51]. The outcome of the effect is mainly dependent on what has been changed in plants caused by the infestation of the herbivore and what are the consequences of these changes for the performance of other organisms [6,52,53]. Therefore, the defense response in plants induced by one herbivore infestation will affect other herbivores differently. For example, an infestation of the defoliator *P. xylostella* but not by the aphid *B. brassicae* attenuates the performance of the root fly *Delia radicum* [21]. Here, we found that an infestation of BPH female adults systemically triggers the JA- and SA-pathway-dependent defenses in rice (Figure 4 and Figure 5). Given that JA signaling plays a central role in regulating rice resistance to herbivores, including SSBs [50] and WBPHs [47], it can be expected that a BPH infestation negatively influences the performance of SSBs and WBPHs. Surprisingly, mutually beneficial relationships between BPHs and SSBs [54,55] and between BPHs and WBPHs [56] have been reported. These consequences are probably because of other signaling pathways systemically activated by a BPH infestation, which jointly regulate the defenses in rice. Therefore, as suggested above, it is necessary to elucidate which signaling pathways regulate the local and systemic defenses in rice induced by a BPH infestation. It should also be noted that the marker genes used in this study—*OsJAZ8*, *OsJAMyb*, *OsPR3*, *OsWRKY62*, *OsPR1a*, and *OsPR10a*—have also been reported to be induced by a pathogen infection and function as important regulators of rice disease resistance [57,58,59,60]. For instance, the overexpression of *OsPR3* and *OsPR1a* enhances the resistance of rice to the sheath blight *Rhizoctonia solani* and leaf blight *Xanthomonas oryzae pv. Oryzae* (*Xoo*), respectively [39,57]. *OsJAZ8*, a repressor of JA-signaling pathways, negatively regulates the resistance to *Xoo* in rice [29], whereas *OsJAMyb*, a JA-responsive transcription factor, positively modulates rice resistance to *Magnaporthe oryza* [61]. Moreover, it has been reported that pre-infestation with BPHs and WBPHs improved rice immunity to the blast fungus *Magnaporthe oryzae* mainly by activating JA and/or SA-mediated signaling pathways [62,63]. Hence, BPH infestation-elicited systemic defenses may also profoundly impact the incidence of rice diseases. Notably, the BPH-induced systemic response is not identical in different rice tissues. The transcript levels of most marker genes in undamaged adjacent leaf blades and sheaths were generally higher than those in distant tissues (Figure 4 and Figure 5). This result may be related to the intensity and transmitting distance of systemic signals. Interestingly, we found that both *OsPR1a* and *OsPR3* showed a higher constitutive transcript level in roots than in other above-ground tissues. Moreover, the transcript level of *OsPR3* increased after the BPH infestation faster in several systemic tissues, especially roots, compared to in local tissues. These results suggest that both *OsPR1a* and *OsPR3*, in addition to their role in regulating the resistance of rice shoots to pathogens as stated above, may also play an important role in regulating the response of rice roots to biotic stresses.

## 5. Conclusions

In this study, we demonstrated that an infestation of gravid BPH females systemically activates JA- and SA-dependent defense responses in rice. These activated systemic responses may influence not only the performance of the BPH itself, but also the performance of other herbivores and pathogens, thereby regulating the composition and structure of the community in the rice ecosystem.

## Data Availability

Not applicable.

## Supplementary Materials

The following supporting information can be downloaded at: https://www.mdpi.com/article/10.3390/biology12060820/s1, Table S1: Primers used for qRT-PCR.
