# Supplementary material for: Infestation by the Piercing–Sucking Herbivore Nilaparvata lugens Systemically Triggers JA- and SA-Dependent Defense Responses in Rice
Source: Biology (Basel). 2023 Jun 5;12(6):820. doi: 10.3390/biology12060820 (PMC10294870; doi:10.3390/biology12060820)
Supplement: Supplementary file 1 [file biology-12-00820-s001.zip › biology-2342758-supplementary.pdf]

Table S1. qRT-PCR primers used in this study.

GENE	LOCUS	F-PRIMER (5'-3')	R-PRIMER (5'-3')
OsHI-LOX	Os08g39840	CCGAGCTTGACGCGAAGA	GATCGTCGTCGTCCACATTGT
OsPAL	Os02g41680	GAGCAGCACAACCAGGACG	CAGAGGGCGATCAAGAACG
OsJAZ8	Os09g26780	GTTACCCACCTCAGCCTCAC	TTTATACGGCGAAACCGAAC
OsWRKY62	Os01g51690	GACGGACAAGATCGCATTCC	TCGAGCACCGGTAGTAGTTC
OsVSP	Os01g09540	ACGCCGGAGCTGAGGAA	TCGAGTTCTCCGACCTTAATATGAG
OsJAMyb	Os11g45740	GTTCACGTCGGAGTCACAAG	CCGTTAAGCTGTTGGTCCTG
OsPR3	Os05g33130	TGGACCTGCTGAGCAACCC	GCGTCATCCAGAACCACAGC
OsPR4	Os11g37970	CATGGTGGTGGCGCTCCT	GATACGTGGCTCGCACGTTG
OsPR1a	Os07g03710	GGTGTCGGAGAAGCAGTGGT	AGGGAGATTGGCCGACGAAG
OsPR1b	Os01g28450	TTGCTTTGGCCATGGTAGCC	CCGGTTGCTGGAGTGGATCA
OsPR10a	Os12g36880	TCCTGTGTGGCCAAGCTCAA	CAGGGTGAGCGACGAGGTAG
OsPR10c	Os03g18850	CAACAGCTGGACCCACGAGA	ATGTGGGAGGCGATCTTGGG
OsACTIN	*Os03g50885*	TGGACAGGTTATCACCATTGGT	CCGCAGCTTCCATTCCTATG
